# Media Exposure, Cancer Beliefs, and Cancer-Related Information-Seeking or Avoidance Behavior Patterns in China

**DOI:** 10.3390/ijerph18063130

**Published:** 2021-03-18

**Authors:** Rui He, Yungeng Li

**Affiliations:** 1 Department of Journalism, School of Humanities, Shanghai University of Finance and Economics, 100 Wudong RD, Yangpu District, Shanghai 200433, China; he.rui@mail.sufe.edu.cn; 2 School of Media and Communication, Shanghai Jiao Tong University, 800 Dongchuan RD, Minhang District, Shanghai 200240, China

**Keywords:** mass and social media exposure, cancer fatalism, cancer curability, cancer-related information seeking, cancer-related information avoidance

## Abstract

This study explored the relationships between media exposure, cancer beliefs, and cancer information-seeking or information-avoidance behaviors. Based on the planned risk information-seeking model and its extended framework, two predictive models were constructed: one for cancer information seeking and the other for cancer information avoidance. A structural equation modeling strategy was applied to survey data from China HINTS 2017 (*n* = 3090) to compare the impact of traditional mass media and social media exposure to cancer-related information on cancer information-seeking and information-avoidance behaviors. The study findings suggest that health-related information exposure through different media channels may generate distinctive information-seeking or information-avoidance behaviors based on various cancer beliefs. Additionally, the findings indicate that social media exposure to health-related and cancer curability beliefs does not lead to cancer information avoidance; both mass media and social media exposure encourage people to seek cancer-related information. Cancer fatalism is positively associated with cancer information-seeking and avoiding intentions, suggesting that negative cancer beliefs predict seemingly contradictory yet psychologically coherent information intentions and behaviors.

## 1. Introduction

Cancer is a severe public health problem in China. In 2018, China ranked first globally for cancer incidence and mortality rate, and the overall cancer incidence and mortality rates in China are increasing [1]. The 5-year average survival rate for cancer patients in China is half that of the United States, Japan, and other developed countries [2]. Medical treatment for cancer patients costs billions of dollars annually and is an economic burden on the state, society, and individuals [3].

Scientific evidence suggests that nearly 40% of cancer cases are preventable [4]. The average cure rate for patients provided with appropriate treatment in the early clinical stages of cancer exceeds 80%. Effective cancer interventions include disseminating preventive knowledge, health education, and behavioral interventions. These interventions aim to raise cancer prevention awareness and control among the Chinese people and monitor and control cancer information [5].

The prevention and treatment of cancer are closely associated with people’s cancer cognition and awareness, which not only relate to scientific knowledge of cancer but also to the risk of developing cancer, risk factors, the effectiveness of treatment, and barriers to seeking medical advice [6,7]. Of these, cancer fatalism is frequently addressed in the literature. In the Western context, cancer fatalism is conceptualized as fatalistic beliefs that hinder people from seeking cancer information and accessing cancer screening and treatment [8]. Consequently, cancer intervention efforts must strengthen cancer prevention awareness and replace fatalistic beliefs with the knowledge that cancer is preventable and curable [9,10]. The cause of cancer is often attributed to “fate”, referring to bad luck or predestination by God [11,12]. However, in Chinese culture, fatalism contains negative and positive elements, and the interplay between the two is complex in shaping the nature and process of cancer-related attitudes and behavior [13].

Cancer information behavior is an essential factor influencing whether an individual adopts a healthy lifestyle or avoids behavior that may increase the risk of developing the disease [14,15]. For instance, cancer fatalism beliefs may facilitate or impede people in seeking treatment information or cancer-related information [16]. However, the scant volumes of empirical evidence have not yet fully unraveled the complexity of psychological mechanisms between health beliefs and cancer-related information behaviors [17].

The media are an external factor that strongly influences the formation or reinforcement of cancer cognition and beliefs [18,19]. The mechanism of media influence on the formation of cancer beliefs and behaviors is incompletely understood. However, cross-sectional and diachronic studies from many countries and regions have indicated that media exposure can increase awareness that cancer is treatable or partially preventable or lead to fatalistic beliefs regarding cancer [20,21]. These seemingly contradictory findings suggest that media exposure can have positive and negative effects in different cultural contexts and among different groups of people [22,23]. Even in the same cultural context, people who use different media types to seek health information may also ascribe different meanings based on their media exposure [24,25,26]. Therefore, it is necessary to distinguish the practical utility of different media for cancer prevention and treatment propaganda. In modern China, social media platforms such as WeChat and Weibo provide a primary health information media environment [5]. Therefore, it is important to explore the functional differences between exposure to social media and other media types in shaping individuals’ cancer cognition and cancer information-seeking behavior.

The influence of media on cancer beliefs and cancer information-related behavior has been examined in academic research and practical intervention [27,28,29]. However, the relationship between media exposure, cancer beliefs, and cancer information-related behavior has not been adequately explored. Previous research has focused on the role of traditional media in cancer beliefs and information seeking. Today, social media play an increasingly significant role in health communication [30]. Therefore, the functional similarities and differences between social media and traditional media as health information sources and influencers on cancer beliefs must be addressed.

## 2. Theoretical Framework and Hypotheses

This paper examines the relationship between health information media exposure, cancer beliefs, and cancer information-seeking or avoidance behavior. Various cognitive models describe the predictors of information seeking or avoidance. The theory of planned behavior (TPB) [31] and the risk information-seeking and processing (RISP) model [32], for instance, are the most frequently mentioned models. To proceed, the planned risk information-seeking model (PRISM), which combines elements from multiple frameworks, provides the most comprehensive perspective.

The PRISM identifies several psychosocial determinants [33], including cognitive and affective motivations [34] that may impact health-related information seeking. The variables concerned with the model are found in the TPB and the RISP and augmented RISP models [35]. The model regards seeking risk information as a planned and active behavior and focuses on variables at the individual level to explain the mechanism of seeking risk information in different contexts. As an integrated model, the PRISM combines the TPB and RISP models, becoming a more suitable initial theoretical model [33].

Of these PRISM variables, the affective factors (e.g., fear and anxiety) play central roles in the need for more information [33]. The model has been applied in fields such as climate change [36], political campaigns [37], health, and risk communication [38]. For example, Hovick expanded the framework by incorporating source beliefs, prior seeking, and results into the PRISM on cancer-related topics [39]. The results show that the PRISM offers a better fit for the data than do the other theoretical models.

Although the PRISM considers an array of influencing psychosocial factors, it does not include the effect of information exposure through the ubiquitous media. Existing studies aim to consider media use in the extended PRISM. According to Ho and colleagues, no significant direct path links media use to seeking intention; the media act as mediators in the PRISM to predict the search for impersonal risk information [40]. However, Niu and colleagues indicate that media use directly predicted information-seeking intention among Chinese groups but not in a U.S. sample [41]. They suggest that the role of media in the PRISM requires further research among diverse geographical or cultural groups.

The mechanism of media use in the PRISM is also consistent with the relationship described in Slater’s reinforcing spirals framework [42]. According to the latter, media use affects various cognitive and behavioral outcomes, which has been addressed in the context of environmental concerns [43]. However, while media use may influence cognition and behavior, it may also result from similar cognition and behavior. For example, information seeking usually involves media use. Both the RISP model and the PRISM suggest that media use is a behavioral outcome of specific cognitions. In combination, these two properties of media use create a series of reinforcement spirals, where the use of media at an early stage influences cognition and behavior, which further impact media use at a later stage. Zhao examined the reinforcing spirals framework in terms of climate-change-related information behaviors. He concluded that media use influenced perceived knowledge, which subsequently directly and indirectly influenced the search for information through environmental concerns [44]. The reinforcing spiral framework outlines the potential for media use to affect beliefs and attitudes over time; current and future media use may reflect current knowledge and cognition [42]. Given its ubiquitous character and complexity, cancer-related information behavior appears an ideal context for examining the interplay between media use and cognition.

Compared to information seeking, the theme of information avoidance received minimal attention. Deline and Kahlor proposed the planned risk avoidance (PRIA) model, which is based on the PRISM but emphasizes the mechanism of risk information avoidance [34]. Avoidance of information is distinct from selecting and searching for information and significant and common information management behaviors [45,46]. It refers to actively avoiding risk-related information [33,36]. The PRIA model suggests that sociocultural factors can lead to cognitive changes, and cognitive factors then lead to affective factors and avoidance (or intentions) of information. However, this framework addresses the impact of cognitive factors on risk information avoidance but not the role of media in cognitive training. Moreover, the assumptions of the model require further data validation.

In summary, the PRISM, together with the extended PRISM, provides theoretical implications for investigating relationships between media, cognitive factors of cancer, and cancer information seeking or avoidance in this study. The PRISM focuses on the relationships between cognition and risk information-seeking intention, and the role of media in the PRISM model has not been fully considered. In comparison, the extended PRISM considers media use; however, the findings on media impacts on information seeking from various studies are inconsistent. The PRIA model, mainly addressing the issue of information-avoidance behavior, is a theoretical hypothesis rather than a normative model and requires further empirical validation. Moreover, most of the prior studies were primarily conducted in the United States, and their applicability to other cultural contexts such as China is unclear. This study starts from the fissures of these studies and aims to comprehend the cancer-related information behaviors in China.

### 2.1. Media Exposure, Cancer Beliefs and Health Information Avoidance

#### 2.1.1. Media Exposure and Cancer Fatalism

It is generally agreed that fatalism is cognitive [13]. It can be conceptualized as a set of beliefs encompassing three dimensions: predestination, pessimism, and attribution of life events to luck [47]. Predestination or fate includes the belief that health is out of one’s control, disease is a punishment for one’s misdeeds, one can do nothing to avoid disease, and everything (including disease) is the will of God [48]. Pessimism includes the belief that death is inevitable once cancer is diagnosed [49,50]; anything can lead to cancer, and one’s risk of cancer cannot be reduced [51]. In the third type of fatalism, good health is attributed to good luck, and poor health (including cancer) is attributed to bad luck [47]. The second dimension of fatalism—pessimism—is widely used in the study of health communication.

Cancer fatalism is a common occurrence. It refers to fears and pessimism that arise in response to the disease [52]. According to the 2003 National Health Information Survey in the United States, about half of the U.S. population believes that cancer is lethal [53]. Individuals of lower socioeconomic status [54], those who belong to ethnic minority groups [55,56], and those with a lower education level [56] are more likely than their peers to believe that cancer is fatal. Asian Americans are more likely than people of other races to express cancer fatalism and are less likely than others to be regularly screened for cancer [57].

Media may influence individuals’ beliefs about the lethality of cancer. Researchers have disputed whether a causal relationship exists between media exposure and a belief that cancer is deadly and, if so, what its direction is [58]. Some studies have considered fatalistic beliefs as a cultural factor influencing media contact. In these cases, the belief that cancer is lethal is considered a unique cultural trait of a particular group, such as African Americans, Latinxs, and Asian Americans. Cultural differences in beliefs about whether cancer is lethal lead to differences in media exposure, reducing health information-seeking behavior among certain groups. However, Mayo et al. found a negative correlation between education and income and lethal beliefs, suggesting that beliefs regarding cancer lethality reflect economic and social disparities rather than racial differences [59]. Moreover, people who report negative information-seeking experiences are more likely to hold fatalistic views about cancer [60].

Other studies have suggested that cancer fatalism may result from media exposure to health information. Numerous cross-sectional and diachronic studies have found that people who are frequently exposed to television tend to have higher levels of fatalistic cancer beliefs than those who do not frequently watch television [61]. The effects of newspaper and radio exposure are inconclusive; these media may reduce or increase fatalistic cancer beliefs [62,63]. Frequent Internet use has not been found to significantly influence or reduce the belief that cancer is fatal [64].

The existing literature has focused on the impact of traditional media on cancer beliefs and behaviors. However, minimal research has examined the effect of social media. Social media platforms, such as Twitter and Facebook, are considered by many to be preferred channels for health information and knowledge [65,66] as well as a source of personal health awareness and health experience. These tools have become platforms for health information monitoring to help people prevent poor health outcomes and intervene in health activities [67]. In China, Weibo and WeChat are common channels by which people access health and cancer information [68]. Therefore, it is critical to analyze the impact of social media on cancer beliefs and information-seeking behavior.

Although no direct evidence exists that social media significantly promote or decrease fatalistic beliefs, studies have shown that the influence of social media on health attitudes is significantly different from that of mass media. Firstly, existing literature suggests that social media have highly interactive Internet features [69]. Since television and print media exposure enhances fatalistic beliefs while Internet exposure diminishes them [68], social media may also reduce cancer fatalism perceptions. Furthermore, social media differ from mass media in that they tend to involve interpersonal communication with local, more personally relevant information [64,70], which is positively associated with lower risk perceptions and a better knowledge of cancer [71,72].

Based on the existing research and theoretical background, the following research hypotheses were proposed:

**Hypothesis** **1A** **(H1A).** *Exposure to health information in the mass media is positively associated with cancer fatalism beliefs.*

**Hypothesis** **1B** **(H1B).** *Exposure to health information on social media is negatively associated with cancer fatalism beliefs.*

#### 2.1.2. Media Exposure and Cancer Curability

The existing research regarding cancer-related beliefs has focused on cancer “fatalism” and “lethality” cognition. Conversely, positive cognition has rarely been discussed as a variable; in some studies, it has been mentioned as part of cancer beliefs [73,74]. Cancer beliefs usually refer to beliefs regarding what causes cancer, the degree to which cancer can be prevented, knowledge of skin cancer risk factors, the severity of cancer, and how long cancer takes to develop [73]. Compared with cancer fatalism, which attributes the cause of cancer to “fate” and emphasizes cancer’s uncertainty, cancer curability stands for positive cognitions that can be measured by agreement with statements such as “Cancer can be cured” or “Cancer can be prevented through a healthy lifestyle, frequent medical check-ups, and early detection.” This study treated the concept of “curability” as an independent factor, reflecting a positive cognition toward cancer.

Generally, exposure to media has been found to reduce negative beliefs about cancer and increase belief in cancer curability. However, this effect varies by media type. For example, Internet exposure has been found to increase the belief that cancer can be cured [66], whereas exposure to television has not been strongly associated with positive cancer beliefs [20]. Similarly, based on these findings, the following hypotheses were proposed:

**Hypothesis** **2A** **(H2A).** *Mass media exposure to health information is negatively associated with cancer curability beliefs.*

**Hypothesis** **2B** **(H2B).** *Social media exposure to health information is positively associated with cancer curability beliefs.*

#### 2.1.3. Cancer Beliefs and Health Information Avoidance

Information avoidance is an important social and psychological research area [75] that includes cognitive and affective factors [76]. Research participants have described cancer information avoidance as a means of control [77], a demonstration of God’s power [78], and a method to reduce anxiety and create hope [79].

Information avoidance is also a selective contact behavior based on cognitive dissonance theory. People avoid information for three main reasons: (a) the information may necessitate a change of belief; (b) the information may necessitate unwanted behavior; (c) the information or one’s decision to learn the information may lead to unpleasant emotions [77]. Therefore, either exposure to cancer information or cancer fatalistic beliefs may lead to information avoidance. People with low self-reported health status and a relatively low education level [80] had difficulty finding appropriate information through new media alone [81] and thus were more likely than others to avoid health information.

Negative and ambiguous beliefs about cancer were found to be barriers to participation in health information browsing, cancer screening, diagnosis, and treatment [82,83,84,85]. The belief that cancer is a “death sentence” (e.g., “When I think of cancer, I think of death”) is a determinant of whether people seek health information. Those who believe that cancer is lethal are likely to develop information-avoidance and treatment-avoidance behaviors [86,87,88,89,90].

Based on the described findings, the following hypothesis was proposed:

**Hypothesis** **3** **(H3).** *Cancer fatalism is positively associated with cancer information avoidance.*

Based on the motivation and mechanism of information avoidance, fear, loss of hope, and insecurity are often essential impetuses for avoiding cancer-related information [77,91]. As a positive belief associated with cancer, curability represents the expectation of hope and is less likely to generate an affective response associated with the mechanism of cancer information avoidance. Therefore, H4 is proposed:

**Hypothesis** **4** **(H4).** *Cancer curability is negatively associated with cancer information avoidance.*

#### 2.1.4. Media Exposure and Cancer Information Avoidance

Media exposure has been found to lead to differing health information intentions and behavior. Exposure to television negatively affects health information browsing and seeking as well as screening for cancer [92]. Relevant research indicates that media reports may cause anxiety and distress, which leads to information avoidance [93], and social media exposure has a significant relationship with information overload and information anxiety [94]. However, other researchers reveal the mechanism between social media and information avoidance, suggesting that social media fatigue rather than social media exposure mediates the impact of information overload on information-avoidance behavior [95,96]. Social media may be a particularly direct medium to effectively communicate information to the public [97] and the interpersonal network [78]. Personal, interpersonal, and social support can assist individuals in managing threatening information and not avoiding health information [98,99,100,101].

Based on the differences between mass media and social media mentioned above, the following hypotheses were proposed:

**Hypothesis** **5A** **(H5A).** *Health information exposure in mass media is positively associated with cancer information avoidance.*

**Hypothesis** **5B** **(H5B).** *Health information exposure in social media is negatively associated with cancer information avoidance.*

### 2.2. Media Exposure, Cancer Beliefs and Cancer Information Seeking

#### 2.2.1. Cancer Beliefs and Cancer Information Seeking

Existing research suggests that people with cancer curability beliefs are more likely than others to actively acquire cancer information [102]. Conversely, individuals with cancer fatalism beliefs are less likely than others to engage in cancer information-seeking or cancer-screening behaviors [103,104]. According to Hay and colleagues, recent Internet searches for health information are associated with cancer curability beliefs [73]. The 2012 China Health Information Trends Survey (2012 China HINTS) also reported that those who believe in cancer curability are more likely than others to seek information about cancer [105].

However, the connection between cancer beliefs and information behavior is complex. Some researchers also noted that the boundaries between information-seeking and information-avoidance behaviors are fluid throughout each patient’s cancer experience [106]. Hopes and fears are intertwined during cancer patients’ treatment, and patients often oscillate between seeking and avoiding information [91]. In some cases, fear of cancer may lead to information-seeking and information-avoidance behaviors, as information can reduce uncertainty or increase anxiety [36,107].

Moreover, as a concept frequently presented as a barrier to cancer control in Western countries, fatalism has an even more complex significance in Chinese culture. The apparently inexplicable nature of cancer, and people’s vulnerability in controlling or changing the cancer outcome, leads many Chinese to adopt fatalistic conclusions. However, fatalism also has a positive aspect in China—the Chinese philosophy of “moushizairen, chengshizaitian” (man proposes, God disposes) encourages people to use various active approaches to address cancer [10,11,57,108]. The interaction between negative interpretation and active coping thereby fosters a culturally unique perspective on fatalism, shaping cancer information processing in complex ways.

Given that this study aimed to explore the relationship between cancer beliefs and cancer information seeking in the Chinese context, the following assumptions were made:

**Hypothesis** **6** **(H6).** *Cancer fatalism is positively associated with cancer information seeking.*

**Hypothesis** **7** **(H7).** *Cancer curability is positively associated with cancer information seeking.*

Although we still posed a negative association between fatalism and information seeking, a more complex relationship between the two was expected and reasonable in the Chinese context.

#### 2.2.2. Media Exposure and Cancer Information Seeking

Past research has found that media exposure—including exposure to health information via newspaper, television, the Internet, and other media sources—can facilitate cancer information seeking [27,79,109]. However, previous studies have focused on the impact of media content and framing on health information-seeking behavior [25] rather than the impact of different types of media exposure on information-seeking behavior.

As noted earlier, studies on the impact of media on health information seeking vary in the extended PRISM. For example, it was demonstrated that media use directly impacted risk perception and affective response but indirectly impacted seeking intention regarding climate change among a Singapore population [40]. Furthermore, media use was directly associated with the intention to seek mental health information in a Chinese sample. Conversely, in a U.S. sample, the effects of media use on information seeking were mediated by subjective norms and perceived knowledge, and no direct effect of media use on seeking intention existed [39]. These studies do not specify the types of media, notwithstanding the varied results.

The media complementarity framework proposed by Dutta-Bergman suggests that the ubiquitous use of new media will not profoundly devastate traditional media use; instead, media consumers will employ the various media as complementary means of information acquisition [27,110]. Henceforth, health information use across various media is not a “zero-sum” process [111]. Instead, people use different media to satisfy diverse needs for health information.

Although the role of media influence in information seeking varies to some extent, different media types may all contribute to it. Thus, the following assumptions were made:

**Hypothesis** **8A** **(H8A).** *Mass media exposure to health information is positively associated with cancer information seeking.*

**Hypothesis** **8B** **(H8B).** *Social media exposure to health information is positively associated with cancer information seeking.*

In summary, this study focused on the relationships between media exposure, cancer beliefs, and cancer information processing, as well as the pathways of influence within these relationships. The differing effects of social media and traditional media on cancer beliefs and cancer information-seeking behaviors were examined, and mechanisms for seeking and avoiding cancer information were explored. In addition to cancer fatalism, positive beliefs about cancer were also considered by introducing cancer curability beliefs as a separate variable in this study.

## 3. Methods and Measurements

### 3.1. Data Source

Data for this paper were drawn from the China Health Information Trends Survey (China HINTS). The instrument for the China survey is borrowed broadly from the U.S. HINTS. However, various adjustments were made to fit its application in China. The first round was held in 2013, and the second was led by researchers from the Beijing Normal University, in collaboration with the China Health Education Center and the China Health Media Group in 2017. Data were collected from residents of Beijing and Hefei. Beijing is the political, economic, and cultural center of China, whereas Hefei is representative of secondary cities in the country. Baseline data on demand for health, cognitive, and behavioral information, and specifically for cancer information, were obtained from the surveys [76,112].

The sample data used in this study are from China HINTS 2017. The survey’s overall target population was all households in Beijing and Hefei, including urban and rural areas. A multistage stratified random sampling methodology was used for the household survey. The district (county), street (township), committee of residence (village), and household were treated as primary, secondary, tertiary, and quaternary sample units, respectively. The household survey had a 64% response rate. The survey fielding occurred from 9–24 May 2017, and the final sample included 3090 respondents.

### 3.2. Measurements

This study explored the relationship between media exposure (mass media and social media), cancer beliefs (cancer fatalism and cancer curability), and cancer information processing (cancer information seeking and cancer information avoidance). The variable indicators were addressed in various parts of the survey questionnaire.

#### 3.2.1. Media Exposure to Health Information

Measurements of media exposure to health information included traditional media and social media exposure. Respondents were asked to report the frequency of their exposure to health or medical topics on various media channels in the previous 12 months on a 4-point scale ranging from “never” to “a lot”. Traditional mass media included newspapers, magazines, radio, and television, whereas social media included Weibo and WeChat, China’s two largest social media platforms (Here, the combined Cronbach coefficient (α = 0.603) of Weibo and WeChat exposure to health information was slightly lower, possibly due to the deviation in the function of people using Weibo and WeChat, wherein Weibo is generally believed to be more information-oriented, while WeChat, on the other hand, is more social. Since the focus of this study was on the differences between social media and mass media in cancer information processing, we did not make a distinction between the two platforms).

#### 3.2.2. Cancer Beliefs

Cancer beliefs were measured in a summated scale adapted from U.S. HINTS. Cancer fatalism was a composite measurement that included respondents’ agreement or disagreement with the following statements: “When I think of cancer, I automatically think of death”; “It seems like almost everything causes cancer”; and “There is not much you can do to lower your chances of getting cancer”. Cancer curability was measured according to respondents’ agreement or disagreement with the following statements: “Cancer is most often caused by a person’s behavior or lifestyle”, “Cancer is an illness that, when detected early, can typically be cured”, “Getting checked regularly for cancer helps find cancer when it’s easy to treat”. Respondents indicated their level of agreement with each statement using a 5-point scale ranging from “strongly disagree” to “strongly agree”.

#### 3.2.3. Cancer Information Processing

Cancer information processing included two indices: cancer information-seeking and cancer information-avoidance behavior. In previous studies, information-seeking behavior has been measured by previous active behavior based on information seeking, information-seeking experiences, and results or information-seeking intention. Previous health information-seeking behavior is a binary variable, which limits the research methodology and analytical results. Information-seeking experiences and results differ based on the individual context; therefore, information-seeking intention has become a key variable for measuring information-seeking behavior. Intention indicates willingness to try and the effort an individual devotes to performing the behavior. Generally, a stronger intention to engage in a behavior is associated with a greater likelihood that the behavior will occur [31] (This has been demonstrated in classical health communication theories, such as the theory of reasonable action (TRA) and the theory of planned behavior (TPB). The integrated model of behavioral prediction (IM) also assumes that the intention to take (or not to take) a measure is a prerequisite for that action. Therefore, information-seeking intention can predict subsequent actual information-seeking behavior). This study used cancer information intention as an indicator of information-seeking behavior.

Respondents’ intention to seek or avoid cancer information was measured by asking them to rate their level of agreement with several statements using a 5-point scale ranging from “strongly disagree” to “strongly agree”. Information-seeking intention included agreement with statements such as “I plan to seek cancer information as much as possible on my initiative” and “I intend to seek cancer information in the near future”. Information-avoidance intention included agreement with statements such as “I worry the cancer information I found will prevent me from dealing with it” and “I always avoid contact with cancer information”.

### 3.3. Analysis Tools

This study explored the relationship between media exposure to health information, cancer beliefs, and cancer information behavior (processing). The analysis relied on certain variables that could not be directly observed. Therefore, structural equation modeling (SEM) was used to detect the relationships among the three groups of variables. SEM was selected for its ability to establish, estimate, and test causality models. It can also be used to analyze the impact of individual indicators on the population and the relationship between individual indicators. Two measurement models were built to account for the fluid boundary between cancer information seeking and avoidance, as previously discussed [113].

## 4. Results

### 4.1. Statistics

The total sample size was 3090 respondents, of which 1527 (49.4%) were located in Beijing and 1563 (50.6%) in Hefei. Of the respondent group, 38.9% were male and 61.1% were female. A detailed statistical description of the results is shown in Table 1.

### 4.2. Structural Equation Modeling (SEM)

In the first step, a measurement model of health beliefs was constructed to conduct confirmative factor analysis. The results are respectively shown in Table 2 and Table 3. Several of the model fit indices were in the excellent range (GFI = 0.998, AGFI = 0.993, NFI = 0.997, CFI = 0.998, RMSEA = 0.027). The ratio of chi-square value to degrees of freedom (x^2^/df = 3.250) was also within an acceptable range. These results indicate that the measurement model was well adjusted. In addition, the composite reliability of both cancer fatalism and curability was higher than 0.6, which indicates excellent reliability. The average variance extracted (AVE) of cancer fatalism was slightly lower than 0.5 while other criteria of excellent convergent and discriminant validity were adequately fulfilled (see note under Table 3). The measurement models shows that the cancer fatalism and cancer curability were two constructs that had adequate reliability and validity (both convergent and discriminant). Therefore, we could use them as comparable constructs in structural models.

Two prediction models were constructed using Amos 24. Model 1 was a structural model for cancer information avoidance, and Model 2 was a structural model for cancer information seeking. There were three latent variables in each model (Model 1: cancer fatalism beliefs, cancer curability beliefs, and cancer information avoidance; Model 2: cancer fatalism beliefs, cancer curability beliefs, and cancer information seeking), and both cancer fatalism and cancer curability beliefs contained three observed variables while both cancer information seeking and avoidance contained two observed variables.

**Figure 1:**
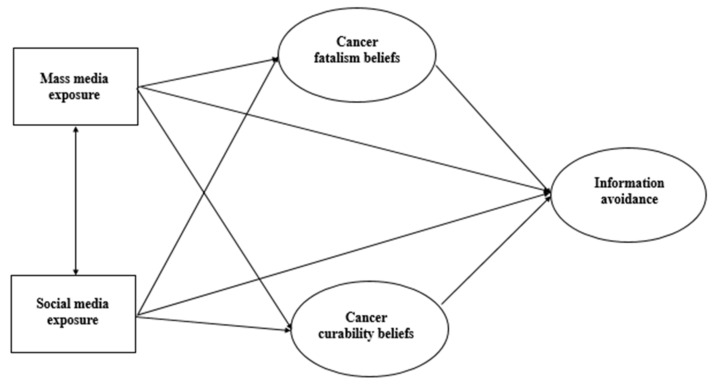
Structural model for cancer information avoidance (Model 1).

**Figure 2:**
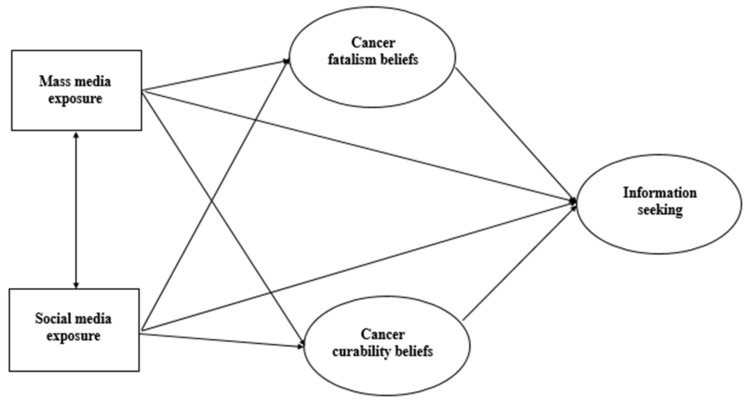
Structural model for cancer information seeking (Model 2).

Two exogenous variables—mass media exposure and social media exposure—were added to construct two prediction models (see Figure 1 and Figure 2). The model was run in Amos using the maximum likelihood method. The co-variation relation between the observed variables’ errors was established according to the modification indices, and two structural equation models of best fit were obtained.

**Figure 3:**
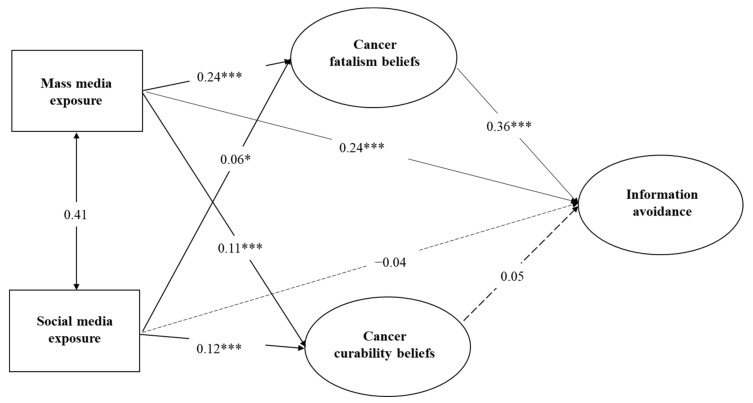
Path analysis of cancer information avoidance (Model 1). Notes * *p* < 0.05, *** *p* < 0.001.

The fit indices for Model 1 are presented in Table 4. Several of these indices were in the excellent range (GFI = 0.996, AGFI = 0.988, NFI = 0.992, CFI = 0.995, RMSEA = 0.028). The ratio of chi-square value to degree-of-freedom (x^2^/df = 3.37) was also within an acceptable range. These results indicate that Model 1 was well adjusted and could account for 25% of the variation in the dependent variable of avoidance of cancer information. Figure 3 presents a detailed path analysis for Model 1. Mass media exposure to health information had positive and significant effects on cancer fatalism beliefs (standardized regression coefficient Beta = 0.24, *p* < 0.001), cancer curability beliefs (standardized Beta = 0.11, *p* < 0.001), and cancer information avoidance (standardized Beta = 0.24, *p* < 0.001). Social media exposure to health information had positive and significant effects on cancer fatalism beliefs (standardized regression coefficient Beta = 0.06, *p* < 0.05), cancer curability beliefs (standardized Beta = 0.12, *p* < 0.001) but a not significant negative effect on information avoidance (standardized Beta = −0.04, *p* > 0.05). Further, cancer fatalism had a significant positive effect on cancer information avoidance (standardized Beta = 0.39, *p* < 0.001) while cancer curability had no significant influence on avoidance (standardized Beta = 0.05, *p* > 0.05). In summary, the study findings based on Model 1 indicate support for hypotheses H1A, H2B, H3, H5A, but not for H1B and H2A, H4, H5B.

**Figure 4:**
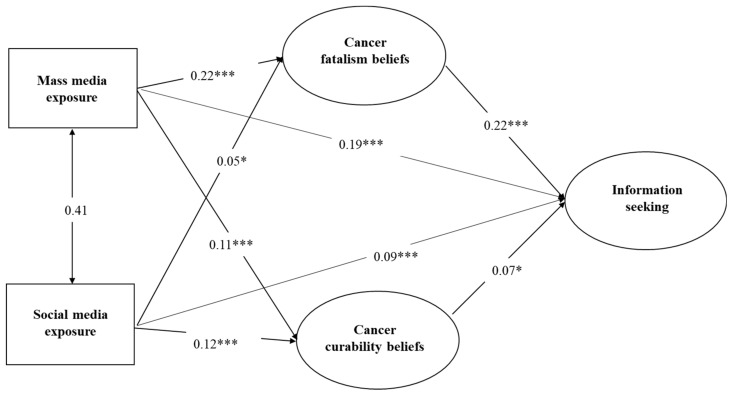
Path analysis of cancer information seeking (Model 2). Notes * *p* < 0.05, *** *p* < 0.001.

The fit indices for Model 2 are presented in Table 5 Several of these indices were in the excellent range (GFI = 0.995, AGFI = 0.986, NFI = 0.992, CFI = 0.994, RMSEA = 0.031). The ratio of chi-square value to degree of freedom (x^2^/df = 3.88) was also within an acceptable range [114]. These results indicate that Model 2 was well adjusted and could account for 16.6% of the variation in the dependent variable of cancer information seeking. Figure 4 presents a detailed path analysis for Model 2. Mass media exposure to health information had positive and significant effects on cancer fatalism (standardized Beta = 0.22, *p* < 0.001), cancer curability beliefs (standardized Beta = 0.11, *p* < 0.001), and cancer information seeking (standardized Beta = 0.19, *p* < 0.001). Social media exposure to health information also had significant positive impacts on cancer fatalism (standardized regression coefficient Beta = 0.05, *p* < 0.05), curability beliefs (standardized Beta = 0.12, *p* < 0.001), and cancer information seeking (standardized Beta = 0.09, *p* < 0.001). Cancer fatalism beliefs had a positive and significant impact on cancer information seeking (standardized Beta = 0.22, *p* < 0.001) and so did curability beliefs (standardized Beta = 0.07, *p* < 0.001). In summary, the study findings based on Model 2 indicate support for hypotheses H6, H7, H8A, and H8B.

## 5. Conclusions and Discussion

### 5.1. Different Roles of Social Media and Mass Media in Influencing Cancer Beliefs and Behaviors

This study validates the importance of the media in influencing individuals’ cancer beliefs and behaviors. Health practice related to cancer must consider the influence of media on the public’s health beliefs and behaviors. Simultaneously, it is important to distinguish between different media types when conducting health promotion and intervention activities.

Within the cancer information-seeking model, mass media and social media differ only slightly. In the cancer information-avoidance model, mass media and social media vary considerably.

#### 5.1.1. The Dual Role of Mass Media in Promoting Cancer Beliefs and Information Behaviors

The study findings indicate that mass media exposure to health information significantly affected positive and negative beliefs about cancer as well as cancer information-seeking and information-avoidance behaviors. Mass media play an important role in providing information and nurturing beliefs. Mass media exposure to health information may promote individuals’ intentions to seek cancer-related information. The act of seeking information usually contributes to the growth of knowledge and implementation of health-related behaviors [112]. Mass media contact can also lead to information avoidance due to poor information quality or information overload. Disinterest in cancer topics and information may also result in information avoidance [115].

Different types of mass media have been associated with either positive or negative cancer perceptions and behaviors. For example, television exposure may increase cancer fatalism beliefs and information-avoidance behavior [20,61,92], whereas newspapers may increase cancer curability beliefs and information-seeking behavior [62,63]. These associations have been found to vary in different cultural settings [24,25,58]. The present study did not distinguish between different types of mass media; however, it suggests that the dual impact of mass media on positive and negative cancer beliefs should be considered in health promotion activities and behavioral interventions.

#### 5.1.2. Social Media Exposure to Health Information Promotes Cancer Curability Beliefs and Cancer Information Seeking

The study results indicate that social media exposure to health information significantly promoted cancer curability beliefs and cancer information-seeking intentions. However, social media exposure to health information did not significantly impact cancer information avoidance. Moreover, its effect on cancer fatalism beliefs was low compared to that of mass media.

Regarding why media channels may lead to different cancer beliefs, a possible explanation is that various media content, such as the form of information and content bias, may affect people’s cancer awareness [29]. In contrast to mass media’s “push” approach to health and cancer information, social media allows users to choose what to focus on. Social media exposure to information also involves more active browsing and seeking behavior by the consumer. The interactive nature of social media and the variety of its content allows users to find answers to personal questions in a timely and multifaceted manner. Furthermore, prior research has suggested that social media users are younger on average than mass media consumers. The proportion of people who use new media declines with age. Specifically, Weibo and WeChat users in China are predominantly younger than 40 [116]. Evidence also exists for more positive and optimistic attitudes toward cancer among youth than among older people.

Considering the characteristics and environment of social media in the health communication process is useful in analyzing the formation of individual beliefs and the intention of information behavior. Promoting communication activities and health interventions can influence people’s willingness to actively seek information about cancer [76].

### 5.2. Cancer Fatalism and Cancer Information Behavior Intervention in Chinese and Western Contexts

It was stimulating to learn that cancer fatalism can increase cancer information seeking and cancer information avoidance. Specifically, cancer fatalism makes people simultaneously seek and avoid cancer information. The standardized effect sizes show that cancer fatalism’s positive relationship with information avoidance was stronger than its relationship with seeking. The conclusions of this study are consistent with previous findings that fatalistic beliefs are a barrier to cancer prevention, control, and intervention among ethnic Chinese. However, they reveal that fatalism may also promote further information seeking under certain circumstances. A survey of Sino-Australian women found that those who believed that disease (including cancer) was unavoidable and could not be prevented had lower screening rates than other groups [117]. In accordance with the study of cancer fear and fatalism [17,89,101,107,118], which is generally understood to be the subset of deterministic attitudes that project a pessimistic rather than optimistic future [51], fatalism may result in negative outcomes in health communication.

However, cancer fatalism is more complex in Chinese culture than in the Western context. The apparently inexplicable nature of cancer and the limited available interventions and ability to change its outcomes have led many Chinese people to believe that cancer is destined and accept that they cannot change its results. However, the Chinese philosophy of “destiny” encourages people to participate in emotional control and self-care activities and take active measures to fight cancer. These conflicting beliefs and resulting behaviors provide a positive application of fatalism beliefs and complicate the processing of cancer information [11,55,119]. In the Chinese context, it is assumed that disseminating culturally appropriate information would reduce public uncertainty, negativity, and external load [55]. Therefore, it is important to compare the influence of cancer beliefs on cancer information behavior in different cultural contexts.

Moreover, studies have suggested that the mechanism by which fatalism functions is by increasing cognitive load or affective risk response. Cognitive load refers to the perception that humans have limited ability to process information. A low cognitive load will increase information avoidance. Affective risk responses, such as fear and worry, may lead to information avoidance. Hence, a distinction must be made between fatalism and other emotions. This remains to be explored further.

The study indicates that cancer curability beliefs facilitate cancer information-seeking intentions and behaviors. Information seeking is often the first step in preventing and treating cancer. These findings suggest that governments and healthcare providers should recognize the different roles of cancer fatalism and curability beliefs when planning health promotion activities and conducting cancer information and behavioral interventions [58,120]. Cancer beliefs and health literacy can also support cancer prevention and control strategies to help people manage cancer crises and reduce the health inequalities associated with socioeconomic status and ethnic diversity [121].

### 5.3. Information Seeking and Avoidance Inclination May Exist Simultaneously

The two models constructed in this study examined the effects of media exposure and cancer beliefs on cancer information behavior. This result indicates that beliefs that cancer is preventable or curable through a healthy lifestyle, physical examinations, and early detection can be evoked by scientific information from mass media or multiplication of information sources through social media. Additionally, the dual, if not contradictory, role of mass media shows that the one-way dissemination of information by mass media may lead to distinct positive or negative health beliefs, which further induce apparently contradictory information behaviors. This is probably because information avoidance and seeking can exist simultaneously, as people who have developed or are afraid of cancer may tend to avoid passive information exposure and simultaneously seek helpful information to reduce anxiety.

Our studies have suggested that both cancer fatalism and cancer curability beliefs may lead to cancer information seeking. For example, information seeking may be driven by positive beliefs in some cases and negative beliefs in others. Conversely, information avoidance may be influenced by negative factors [99]. However, this conclusion is not final; in the cancer information process, the line between information seeking and avoidance is not stationary [115]. Research has also indicated that disinterest, wherein people believe that cancer is irrelevant to them, differs from information avoidance [119]. Further investigation is required to examine the effect of disinterest on cancer information behavior.

## 6. Limitations

This study has several limitations. First, it examined the relationships between media exposure to health information, cancer beliefs, and cancer information behavior. However, other variables including demographic variables such as gender and age also influence public beliefs about cancer. The population sample from which this study’s data were drawn was disproportionately skewed toward young people, who primarily use social media to access health information. Conversely, the proportion of seniors in the sample was relatively small. Future research should expand on this study to examine demographic factors, including similarities and differences in the influence of media use on seniors.

Second, media exposure to health information was used to connect cancer beliefs with cancer information behavior and compare similarities and differences between mass media exposure and social media exposure. This division of media was verified in the two models built in this study and therefore has some explanatory power. However, the model used in this study did not distinguish between the different types of mass media. Additionally, other researchers have proposed that the construction of cancer issues in media influences public perceptions, attitudes, and behaviors toward cancer. Therefore, measuring other media use dimensions (e.g., news content consumption and active media use) may increase confidence in the present study’s interpretations.

Third, the study findings are not entirely consistent with research conclusions about the impact of cancer fatalism beliefs on cancer information behavior in the Western context. In Chinese culture, cancer fatalism beliefs are widely held and are often associated with ancient philosophy, traditional Chinese medicine, and other cultural traditions. Additional research is required to determine the influence of such beliefs on cancer information processing in the Chinese context.

Finally, this study examined the relationships between media exposure to health information, cancer beliefs, and cancer information behavior in the past 12 months, based on cross-sectional data. The study models are somewhat convincing; however, the formation and change of beliefs and behaviors may be a long-term and subtle process that could be verified and further described by future research experiments or diachronic studies.

## Figures and Tables

**Table 1:** Sample descriptive statistics (*N* = 3090).

Type	Variable	Categories	Percentage or Mean (SD)
Cancer Information Behaviors (strongly disagree (1)–strongly agree (5))	Cancer information seeking (intention)	I plan to seek cancer information as much as possible on my initiative	2.65 (1.052)
		I intend to seek cancer information in the near future	2.68 (1.059)
	Cancer information avoidance (intention)	I worry the cancer information I found will prevent me from dealing with it	2.86 (1.000)
		I always avoid contact with cancer information	2.75 (0.999)
Media Exposure to Health Information (never (1)–a lot (4))	(Traditional) Mass media exposure to health information (4 items, α = 0.814)	Newspapers	2.01 (0.756)
		Magazines	
		Radio	
		Television	
	Social media exposure to health information (2 items, α = 0.603)	Weibo	2.27 (0.858)
		WeChat	
Cancer Beliefs (strongly disagree (1)–strongly agree (5))	Cancer fatalism	When I think of cancer, I automatically think of death	2.94 (1.149)
		It seems like almost everything causes cancer	3.32 (1.066)
		There is not much you can do to lower your chances of getting cancer	2.94 (1.044)
	Cancer curability	Cancer is most often caused by a person’s behavior or lifestyle	3.27 (1.010)
		Getting checked regularly for cancer helps find cancer when it’s easy to treat	3.57 (0.999)
		Cancer is an illness that, when detected early, can typically be cured	3.49 (1.005)
Demographics	Gender	Male	38.9%
		Female	61.1%
	Age	18–24	19.1%
		25–39	48.2%
		40–59	29.8%
		≥60	2.8%
	Ethnicity	Han	98.7%
		Minority	1.3%
	Education	Less than high school	18.0%
		High school	27.0%
		Vocational school	26.1%
		College and above	28.9%
	Family Income	¥50,000 and less	26.7%
		¥50,001–¥100,000	38.2%
		¥100,001–¥200,000	23.3%
		¥200,001 and above	11.8%
	Marital Status	Married	70.6%
		Not married	29.4%

**Table 2:** Fit indices for measurement model of health beliefs.

Model Fit			Absolute Fit Indices			Incremental Fit Indices			Parsimony Fit Indices	
	X^2^	df	X^2^/df	GFI	RMSEA	NFI	CFI	PNFI	AGFI	PGFI
Good			<3	>0.90	<0.05	>0.90	>0.90	>0.80	>0.90	>0.80
Acceptable			<5	>0.70	<0.08	>0.70	>0.70	>0.50	>0.70	>0.50
Default Model	16.248	5	3.250	0.998	0.027	0.997	0.998	0.332	0.993	0.238

**Table 3:** Constructs reliability and validity.

Constructs (Health Beliefs)	CR	AVE	MSV	Correlations (CF)	Correlations (CC)
Cancer Fatalism (CF)	0.682	0.428	0.320	1	0.647 ***
Cancer Curability (CC)	0.808	0.588		0.647 ***	1

Note: CR: composite reliability; AVE: average variance extracted; MSV: maximum shared variance. Acceptable convergent validities indicate that the following criteria were met: for each construct (a) the composite reliability was greater than 0.50; (b) the square root of average variance extracted (AVE) was larger than 0.50; (c) the composite reliability was larger than AVE; an acceptable discriminant validity for each construct means that the AVE was greater than the maximum shared variance (MSV). *** *p* < 0.001.

**Table 4:** Fit indices for Model 1.

Model Fit			Absolute Fit Indices			Incremental Fit Indices			Parsimony Fit Indices	
	X^2^	df	X^2^/df	GFI	RMSEA	NFI	CFI	PNFI	AGFI	PGFI
Good			<3	>0.90	<0.05	>0.90	>0.90	>0.80	>0.90	>0.80
Acceptable			<5	>0.70	<0.08	>0.70	>0.70	>0.50	>0.70	>0.50
Default Model	67.35	20	3.37	0.996	0.028	0.992	0.995	0.441	0.988	0.362

**Table 5:** Fit indices for Model 2.

Model Fit			Absolute Fit Indices			Incremental Fit Indices			Parsimony Fit Indices	
	X^2^	df	X^2^/df	GFI	RMSEA	NFI	CFI	PNFI	AGFI	PGFI
Good			<3	>0.90	<0.05	>0.90	>0.90	>0.80	>0.90	>0.80
Acceptable			<5	>0.70	<0.08	>0.70	>0.70	>0.50	>0.70	>0.50
Default Model	81.52	21	3.88	0.995	0.031	0.992	0.994	0.463	0.986	0.380

## Data Availability

Restrictions apply to the availability of these data. Data was obtained from Beijing Normal University and China Health Media Group and are available with the permission of Yu Guoming Team of Beijing Normal University.

## References

[1] Bray F., Ferlay J., Soerjomataram I., Siegel R.L., Torre L.A., Jemal A. (2018). Global cancer statistics 2018: GLOBOCAN estimates of incidence and mortality worldwide for 36 cancers in 185 countries. CA Cancer J. Clin..

[2] Allemani C., Matsuda T., Di Carlo V., Harewood R., Matz M., Nikšić M., Bonaventure A., Valkov M., Johnson C.J., Estève J. (2018). Global surveillance of trends in cancer survival 2000–14 (CONCORD-3): Analysis of individual records for 37 513025 patients diagnosed with one of 18 cancers from 322 population-based registries in 71 countries. Lancet.

[3] An Interpretation of the Implementation Plan for Cancer Prevention and Treatment in Healthy China Action (2019–2022). http://www.xinhuanet.com/health/2019-09/23/c_1125029208.htm.

[4] World Cancer Research Fund & American Institute for Cancer Research Diet, Nutrition, Physical Activity and Cancer: A Global Perspective (The Third Expert Report). https://www.wcrf.org/sites/default/files/Summary-of-Third-Expert-Report-2018.pdf.

[5] Chinese Center for Disease Control and Prevention The Three-Year Action Plan for Cancer Prevention and Control in China. http://www.nhc.gov.cn/jkj/s5878/201509/656437bc5c7e4cd0afb581de85be998a.shtml.

[6] Austoker J., Bankhead C., Forbes L., Atkins L., Martin F., Robb K., Wardle J., Ramirez A. (2009). Interventions to promote cancer awareness and early presentation: Systematic review. Nat. Preced..

[7] Stubbings S., Robb K., Waller J., Ramirez A., Austoker J., Macleod U., Hiom S., Wardle J. (2009). Development of a measurement tool to assess public awareness of cancer. Br. J. Cancer.

[8] Goss P.E., Strasser-Weippl K., Lee-Bychkovsky B.L., Fan L., Li J., Chavarri-Guerra Y., Liedke P.E., Pramesh C.S., Badovinac-Crnjevic T., Sheikine Y. (2014). Challenges to effective cancer control in China, India, and Russia. Lancet Oncol..

[9] Liang W., Yuan E., Mandelblatt J.S., Pasick R.J. (2004). How do older Chinese women view health and cancer screening? Results from focus groups and implications for interventions. Ethn. Health.

[10] Heiniger L.E., Sherman K.A., Shaw L.K.E., Costa D. (2015). Fatalism and health promoting behaviors in Chinese and Korean immigrants and Caucasians. J. Immigr. Minority Health.

[11] Cheng H., Sit J.W., Twinn S.F., Cheng K.K., Thorne S. (2013). Coping with breast cancer survivorship in Chinese women: The role of fatalism or fatalistic voluntarism. Cancer Nurs..

[12] Xie J., Xie S., Cheng Y., He Z. (2020). Beliefs and Information Seeking in Patients with Cancer in Southwest China: Survey Study. Jmir Cancer.

[13] Tong X. (2017). Fatalism and the Processing of Fear Appeals among Chinese: An Exploratory Study in the Context of Lung Cancer Prevention. Ph.D. Thesis.

[14] Shim M., Kelly B., Hornik R. (2006). Cancer information scanning and seeking behavior is associated with knowledge, lifestyle choices, and screening. J. Health Commun..

[15] Ramírez A.S., Freres D., Martinez L.S., Lewis N., Bourgoin A., Kelly B.J., Lee C.J., Nagler R., Schwartz J.S., Hornik R.C. (2013). Information seeking from media and family/friends increases the likelihood of engaging in healthy lifestyle behaviors. J. Health Commun..

[16] Kim H.K., Lwin M.O. (2020). Cultural Determinants of Cancer Fatalism and Cancer Prevention Behaviors among Asians in Singapore. Health Commun..

[17] Chae J. (2016). Who avoids cancer information? Examining a psychological process leading to cancer ceance. J. Health Commun..

[18] Oakley A., Bendelow G., Barnes J., Buchanan M., Husain O.N. (1995). Health and cancer prevention: Knowledge and beliefs of children and young people. BMJ.

[19] Wong-Kim E., Sun A., DeMattos M.C. (2003). Assessing cancer beliefs in a Chinese immigrant community. Cancer Control.

[20] Niederdeppe J., Fowler E.F., Goldstein K., Pribble J. (2010). Does local television news coverage cultivate fatalistic beliefs about cancer prevention?. J. Commun..

[21] Im H., Huh J. (2017). Does health information in mass media help or hurt patients? Investigation of potential negative influence of mass media health information on patients’ beliefs and medication regimen adherence. J. Health Commun..

[22] Lee C.J., Niederdeppe J., Freres D. (2012). Socioeconomic disparities in fatalistic beliefs about cancer prevention and the Internet. J. Commun..

[23] Somera L.P., Badowski G., Cassell K., Lee H.R. Health information sources among Pacific Islanders in Guam and Hawaii: The association of migrant status and acculturation with Internet use and cancer fatalism. Proceedings of the Twelfth AACR Conference on the Science of Cancer Health Disparities in Racial/Ethnic Minorities and the Medically Underserved.

[24] Befort C.A., Nazir N., Engelman K., Choi W. (2013). Fatalistic cancer beliefs and information sources among rural and urban adults in the USA. J. Cancer Educ..

[25] Jensen J.D., Carcioppolo N., King A.J., Bernat J.K., Davis L., Yale R., Smith J. (2011). Including limitations in news coverage of cancer research: Effects of news hedging on fatalism, medical skepticism, patient trust, and backlash. J. Health Commun..

[26] Sinky T.H., Faith J., Lindly O., Thorburn S. (2018). Cancer fatalism and preferred sources of cancer information: An assessment using 2012 hints data. J. Cancer Educ..

[27] Tian Y., Robinson J.D. (2008). Media use and health information seeking: An empirical test of complementarity theory. Health Commun..

[28] Oh K.M., Kreps G.L., Jun J., Ramsey L. (2011). Cancer information seeking and awareness of cancer information sources among Korean Americans. J. Cancer Educ..

[29] Nagler R.H., Hornik R.C. (2012). Measuring media exposure to contradictory health information: A comparative analysis of four potential measures. Commun. Methods Meas..

[30] Lumpkins C.Y., Mabachi N., Lee J., Pacheco C., Greiner K.A., Geana M. (2017). A prescription for internet access: Appealing to middle-aged and older racial and ethnic minorities through social network sites to combat colorectal cancer. Health Commun..

[31] Ajzen I. (1991). The theory of planned behavior. Organ. Behav. Hum. Decis. Process..

[32] Dunwoody S., Griffin R.J. (2015). Risk information seeking and processing model. The SAGE Handbook of Risk Communication.

[33] Kahlor L. (2010). PRISM: A planned risk information seeking model. Health Commun..

[34] Deline M.B., Kahlor L.A. (2019). Planned risk information avoidance: A proposed theoretical model. Commun. Theory.

[35] Kahlor L.A. (2007). An augmented risk information seeking model: The case of global warming. Media Psychol..

[36] Yang Z.J., Kahlor L. (2013). What, me worry? The role of affect in information seeking and avoidance. Sci. Commun..

[37] Kahlor L.A., Yang Z.J., Liang M.C. (2018). Risky politics: Applying the planned risk information seeking model to the 2016 US presidential election. Mass Commun. Soc..

[38] Hubner A.Y., Hovick S.R. (2020). Understanding risk information seeking and processing during an infectious disease outbreak: The case of Zika virus. Risk Anal..

[39] Hovick S.R., Kahlor L., Liang M.C. (2014). Personal cancer knowledge and information seeking through PRISM: The planned risk information seeking model. J. Health Commun..

[40] Ho S.S., Detenber B.H., Rosenthal S., Lee E.W. (2014). Seeking information about climate change: Effects of media use in an extended PRISM. Sci. Commun..

[41] Niu Z., Willoughby J.F., Mei J., Li S., Hu P. (2020). A cross-cultural comparison of an extended planned risk information seeking model on mental health among college students: Cross-sectional study. J. Med. Internet Res..

[42] Slater M.D. (2007). Reinforcing spirals: The mutual influence of media selectivity and media effects and their impact on individual behavior and social identity. Commun. Theory.

[43] Zhao X. (2012). Personal values and environmental concern in China and the US: The mediating role of informational media use. Commun. Monogr..

[44] Zhao X. (2009). Media use and global warming perceptions: A snapshot of the reinforcing spirals. Commun. Res..

[45] Narayan B., Case D.O., Edwards S.L. (2011). The role of information avoidance in everyday-life information behaviors. Proc. Am. Soc. Inf. Sci. Technol..

[46] Barbour J.B., Rintamaki L.S., Ramsey J.A., Brashers D.E. (2012). Avoiding health information. J. Health Commun..

[47] Shen L., Condit C.M. (2011). Addressing fatalism with health messages. Health Communication Message Design: Theory and Practice.

[48] Straughan P.T., Seow A. (1998). Fatalism conceptualized: A concept to predict health screening behavior. J. Gend. Cult. Health..

[49] Powe B.D. (2001). Cancer fatalism among elderly African American women: Predictors of the intensity of the perceptions. J. Psychosoc. Oncol..

[50] Powe B.D., Finnie R. (2003). Cancer fatalism: The state of the science. Cancer Nurs..

[51] Keeley B., Wright L., Condit C.M. (2009). Functions of health fatalism: Fatalistic talk as face saving, uncertainty management, stress relief and sense making. Sociol. Health Illn..

[52] Lawsin C., DuHamel K., Weiss A., Rakowski W., Jandorf L. (2007). Colorectal cancer screening among low-income African Americans in East Harlem: A theoretical approach to understanding barriers and promoters to screening. J. Urban Health.

[53] Niederdeppe J., Levy A.G. (2007). Fatalistic beliefs about cancer prevention and three prevention behaviors. Cancer Epidemiol. Prev. Biomark..

[54] Vanderpool R.C., Huang B. (2010). Cancer risk perceptions, beliefs, and physician avoidance in Appalachia: Results from the 2008 HINTS Survey. J. Health Commun..

[55] Vrinten C., Wardle J., Marlow L.A. (2016). Cancer fear and fatalism among ethnic minority women in the United Kingdom. Br. J. Cancer.

[56] Ramírez A.S., Rutten L.J.F., Oh A., Vengoechea B.L., Moser R.P., Vanderpool R.C., Hesse B.W. (2013). Perceptions of cancer controllability and cancer risk knowledge: The moderating role of race, ethnicity, and acculturation. J. Cancer Educ..

[57] Jun J., Oh K.M. (2013). Asian and Hispanic Americans’ cancer fatalism and colon cancer screening. Am. J. Health Behav..

[58] Ramondt S., Ramírez A.S. (2017). Fatalism and exposure to health information from the media: Examining the evidence for causal influence. Ann. Int. Commun. Assoc..

[59] Mayo R.M., Ureda J.R., Parker V.G. (2001). Importance of fatalism in understanding mammography screening in rural elderly women. J. Women Aging.

[60] Arora N.K., Hesse B.W., Rimer B.K., Viswanath K., Clayman M.L., Croyle R.T. (2008). Frustrated and confused: The American public rates its cancer-related information-seeking experiences. J. Gen. Intern. Med..

[61] Lee C.J., Niederdeppe J. (2011). Genre-specific cultivation effects: Lagged associations between overall TV viewing, local TV news viewing, and fatalistic beliefs about cancer prevention. Commun. Res..

[62] Chung J.E. (2014). Medical dramas and viewer perception of health: Testing cultivation effects. Hum. Commun. Res..

[63] Kealey E., Berkman C.S. (2010). The relationship between health information sources and mental models of cancer: Findings from the 2005 Health Information National Trends Survey. J. Health Commun..

[64] Han R., Cheng Y. (2020). The influence of norm perception on pro-environmental behavior: A comparison between the moderating roles of traditional media and social Media. Int. J. Environ. Res. Public Health.

[65] Lunsford N.B., Berktold J., Holman D.M., Stein K., Prempeh A., Yerkes A. (2018). Skin cancer knowledge, awareness, beliefs and preventive behaviors among black and hispanic men and women. Prev. Med. Rep..

[66] Groshek J., Katz J.E., Andersen B., Cutino C., Zhong Q. (2018). Media use and antimicrobial resistance misinformation and misuse: Survey evidence of information channels and fatalism in augmenting a global health threat. Cogent Med..

[67] Chew C., Eysenbach G. (2010). Pandemics in the age of Twitter: Content analysis of Tweets during the 2009 H1N1 outbreak. PLoS ONE.

[68] Yu G., Pan J., Kreps G. (2017). The norms of health communication research: Theoretical framework and academic logic based on the Chinese health information national trends survey. Editor. Friend.

[69] Ho S.S., Liao Y., Rosenthal S. (2015). Applying the theory of planned behavior and media dependency theory: Predictors of public pro-environmental behavioral intentions in Singapore. Environ. Commun..

[70] Oh O., Agrawal M., Rao H.R. (2013). Community intelligence and social media services: A rumor theoretic analysis of tweets during social crises. Mis Q..

[71] Lee E.W., Ho S.S., Chow J.K., Wu Y.Y., Yang Z. (2013). Communication and knowledge as motivators: Understanding Singaporean women’s perceived risks of breast cancer and intentions to engage in preventive measures. J. Risk Res..

[72] Lee E.W., Ho S.S. (2015). Staying abreast of breast cancer: Examining how communication and motivation relate to Singaporean women’s breast cancer knowledge. Asian J. Commun..

[73] Hay J., Coups E.J., Ford J., DiBonaventura M. (2009). Exposure to mass media health information, skin cancer beliefs, and sun protection behaviors in a United States probability sample. J. Am. Acad. Dermatol..

[74] Donnelly T.T., Al Khater A.H., Al-Bader S.B., Al Kuwari M.G., Al-Meer N., Malik M., Singh R., Chaudhry S., Fung T. (2013). Beliefs and attitudes about breast cancer and screening practices among Arab women living in Qatar: A cross-sectional study. BMC Women’s Health.

[75] Case D.O., Andrews J.E., Johnson J.D., Allard S.L. (2005). Avoiding versus seeking: The relationship of information seeking to avoidance, blunting, coping, dissonance, and related concepts. J. Med. Libr. Assoc..

[76] Niederdeppe J., Hornik R.C., Kelly B.J., Frosch D.L., Romantan A., Stevens R.S., Barg F.K., Weiner J.L., Schwartz J.S. (2007). Examining the dimensions of cancer-related information seeking and scanning behavior. Health Commun..

[77] Sweeny K., Melnyk D., Miller W., Shepperd J.A. (2010). Information avoidance: Who, what, when, and why. Rev. Gen. Psychol..

[78] Costas-Muniz R., Sen R., Leng J., Aragones A., Ramirez J., Gany F. (2013). Cancer stage knowledge and desire for information: Mismatch in Latino cancer patients?. J. Cancer Educ..

[79] Mayer D.K., Terrin N.C., Kreps G.L., Menon U., McCance K., Parsons S.K., Mooney K.H. (2007). Cancer survivors’ information seeking behaviors: A comparison of survivors who do and do not seek information about cancer. Patient Educ. Couns..

[80] Jung M., Ramanadhan S., Viswanath K. (2013). Effect of information seeking and avoidance behavior on self-rated health status among cancer survivors. Patient Educ. Couns..

[81] McCloud R.F., Jung M., Gray S.W., Viswanath K. (2013). Class, race and ethnicity and information avoidance among cancer survivors. Br. J. Cancer.

[82] Emanuel A.S., Kiviniemi M.T., Howell J.L., Hay J.L., Waters E.A., Orom H., Shepperd J.A. (2015). Avoiding cancer risk information. Soc. Sci. Med..

[83] Ramírez A.S. (2014). Fatalism and cancer risk knowledge among a sample of highly acculturated Latinas. J. Cancer Educ..

[84] Smith-Howell E.R., Rawl S.M., Champion V.L., Skinner C.S., Springston J., Krier C., Russell K.M., Perkins S., Rhyant B., Lloyd F. (2011). Exploring the role of cancer fatalism as a barrier to colorectal cancer screening. West. J. Nurs. Res..

[85] Baumann E., Scherer H., Link E., Wiltfang J., Wenz H.J., Koller M., Hertrampf K. (2019). Exploratory Research Focusing on Oral Cancer Prevention: Challenges of Dealing With Informational and Cognitive Barriers. Qual. Health Res..

[86] Moser R.P., Arndt J., Han P.K., Waters E.A., Amsellem M., Hesse B.W. (2014). Perceptions of cancer as a death sentence: Prevalence and consequences. J. Health Psychol..

[87] Morris N.S., Field T.S., Wagner J.L., Cutrona S.L., Roblin D.W., Gaglio B., Williams A.E., Han P.J.K., Costanza M.E., Mazor K.M. (2013). The association between health literacy and cancer-related attitudes, behaviors, and knowledge. J. Health Commun..

[88] Kannan V.D., Veazie P.J. (2014). Predictors of avoiding medical care and reasons for avoidance behavior. Med. Care.

[89] Miles A., Voorwinden S., Chapman S., Wardle J. (2008). Psychologic predictors of cancer information avoidance among older adults: The role of cancer fear and fatalism. Cancer Epidemiol. Biomark. Prev..

[90] Persoskie A., Ferrer R.A., Klein W.M. (2014). Association of cancer worry and perceived risk with doctor avoidance: An analysis of information avoidance in a nationally representative US sample. J. Behav. Med..

[91] Leydon G.M., Boulton M., Moynihan C., Jones A., Mossman J., Boudioni M., McPherson K. (2000). Cancer patients’ information needs and information seeking behaviour: In depth interview study. BMJ.

[92] Han P.K., Moser R.P., Klein W.M., Beckjord E.B., Dunlavy A.C., Hesse B.W. (2009). Predictors of perceived ambiguity about cancer prevention recommendations: Sociodemographic factors and mass media exposures. Health Commun..

[93] Siebenhaar K.U., Köther A.K., Alpers G.W. (2020). Dealing with the COVID-19 infodemic: Distress by information, information avoidance, and compliance with preventive measures. Front. Psychol..

[94] Soroya S.H., Farooq A., Mahmood K., Isoaho J., Zara S.E. (2020). From information seeking to information avoidance: Understanding the health information behavior during a global health crisis. Inf. Process. Manag..

[95] Dai B., Ali A., Wang H. (2020). Exploring information avoidance intention of social media users: A cognition–affect–conation perspective. Internet Res..

[96] Guo Y., Lu Z., Kuang H., Wang C. (2020). Information avoidance behavior on social network sites: Information irrelevance, overload, and the moderating role of time pressure. Int. J. Inf. Manag..

[97] Lachlan K.A., Spence P.R., Lin X. (2014). Expressions of risk awareness and concern through Twitter: On the utility of using the medium as an indication of audience needs. Comput. Hum. Behav..

[98] Howell J.L., Crosier B.S., Shepperd J.A. (2014). Does lacking threat-management resources increase information avoidance? A multi-sample, multi-method investigation. J. Res. Personal..

[99] Howell J.L., Shepperd J.A. (2017). Social exclusion, self-affirmation, and health information avoidance. J. Exp. Soc. Psychol..

[100] Howell J.L., Lipsey N.P., Shepperd J.A. (2020). Health information avoidance. Wiley Encycl. Health Psychol..

[101] Chae J., Lee C.J., Kim K. (2020). Prevalence, predictors, and psychosocial mechanism of cancer information avoidance: Findings from a national survey of US adults. Health Commun..

[102] Kaphingst K.A., Lachance C.R., Condit C.M. (2009). Beliefs about heritability of cancer and health information seeking and preventive behaviors. J. Cancer Educ..

[103] Miles A., Rainbow S., von Wagner C. (2011). Cancer fatalism and poor self-rated health mediate the association between socioeconomic status and uptake of colorectal cancer screening in England. Cancer Epidemiol. Prev. Biomark..

[104] Azaiza F., Cohen M., Awad M., Daoud F. (2010). Factors associated with low screening for breast cancer in the Palestinian authority: Relations of availability, environmental barriers, and cancer-related fatalism. Cancer.

[105] Zhao X., Mao Q., Kreps G.L., Yu G., Li Y., Chou S.W.-Y., Perkosie A., Nie X., Xu Z., Song M. (2015). Cancer information seekers in China: A preliminary profile. J. Health Commun..

[106] Germeni E., Schulz P.J. (2014). Information seeking and avoidance throughout the cancer patient journey: Two sides of the same coin? A synthesis of qualitative studies. Psycho-Oncology.

[107] Chae J. (2015). A three-factor cancer-related mental condition model and its relationship with cancer information use, cancer information avoidance, and screening intention. J. Health Commun..

[108] Lo G., Chen J., Wasser T., Portenoy R., Dhingra L. (2016). Initial validation of the Daily Spiritual Experiences Scale in Chinese immigrants with cancer pain. J. Pain Symptom Manag..

[109] Portnoy D.B., Leach C.R., Kaufman A.R., Moser R.P., Alfano C.M. (2014). Reduced fatalism and increased prevention behavior after two high-profile lung cancer events. J. Health Commun..

[110] Dutta-Bergman M.J. (2004). Complementarity in consumption of news types across traditional and new media. J. Broadcasting Electron. Media.

[111] Griswold W., Wright N., Howard P.N., Jones S. (2004). Wired and well read. Society Online: The Internet in Context.

[112] Johnson J.D., Meischke H. (1993). A comprehensive model of cancer-related information seeking applied to magazines. Hum. Commun. Res..

[113] Anglim J. Structural Equation Modeling. http://web.psych.unimelb.edu.au/jkanglim/IntroductiontoSEM.pdf.

[114] Wu M.L. (2009). Structural Equation Model: AMOS Operations and Applications.

[115] Lambert S.D., Loiselle C.G., Macdonald M.E. (2009). An in-depth exploration of information-seeking behavior among individuals with cancer: Part 2: Understanding patterns of information disinterest and avoidance. Cancer Nurs..

[116] He L.N., Zhang Z.A., Lee W., Lai K.S. (2016). User behavior and motivation of two micro-terminals. Media.

[117] Kwok C., Sullivan G. (2006). Influence of traditional Chinese beliefs on cancer screening behaviour among Chinese-Australian women. J. Adv. Nurs..

[118] Leung D.Y., Chow T.T., Wong E.M. (2017). Cancer-Related Information Seeking and Scanning Behaviors among Older Chinese Adults: Examining the Roles of Fatalistic Beliefs and Fear. Geriatrics.

[119] Kim H.K., Lwin M.O. (2017). Cultural effects on cancer prevention behaviors: Fatalistic cancer beliefs and risk optimism among Asians in Singapore. Health Commun..

[120] Powe B.D., Johnson A. (1995). Fatalism as a barrier to cancer screening among African-Americans: Philosophical perspectives. J. Relig. Health.

[121] Kobayashi L.C., Smith S.G. (2016). Cancer fatalism, literacy, and cancer information seeking in the American public. Health Educ. Behav..

